# Mental health care providers' suggestions for suicide prevention among people with substance use disorders in South Africa: a qualitative study

**DOI:** 10.1186/s13011-018-0185-y

**Published:** 2018-12-07

**Authors:** Daniel Goldstone, Jason Bantjes, Lisa Dannatt

**Affiliations:** 1 Department of Psychology, Stellenbosch University, Private Bag X1, Matieland, Stellenbosch, 7602 South Africa; 2 Department of Psychiatry and Mental Health, J-Block, Groote Schuur Hospital, Observatory, Cape Town, South Africa

**Keywords:** South Africa, Substance use disorder, Suicide prevention, Low- and middle-income country, Qualitative research

## Abstract

**Background:**

People with substance use disorders (PWSUDs) are a clearly delineated group at high risk for suicidal behaviour. Expert consensus is that suicide prevention strategies should be culturally sensitive and specific to particular populations and socio-cultural and economic contexts. The aim of this study was to explore mental health care providers' context- and population-specific suggestions for suicide prevention when providing services for PWSUDs in the Western Cape, South Africa.

**Methods:**

Qualitative data were collected via in-depth, semi-structured interviews with 18 mental health care providers providing services to PWSUDs in the public and private health care sectors of the Western Cape, South Africa. Data were analysed inductively using thematic analysis.

**Results:**

Participants highlighted the importance of providing effective mental health care, transforming the mental health care system, community interventions, and early intervention, in order to prevent suicide amongst PWSUDs. Many of their suggestions reflected basic principles of effective mental health care provision. However, participants also suggested further training in suicide prevention for mental health care providers, optimising the use of existing health care resources, expanding service provision for suicidal PWSUDs, improving policies and regulations for the treatment of substance use disorders, provision of integrated health care, and focusing on early intervention to prevent suicide.

**Conclusions:**

Training mental health care providers in suicide prevention must be augmented by addressing systemic problems in the provision of mental health care and contextual problems that make suicide prevention challenging. Many of the suggestions offered by these participants depart from individualist, biomedical approaches to suicide prevention to include a more contextual view of suicide prevention. A re-thinking of traditional bio-medical approaches to suicide prevention may be warranted in order to reduce suicide among PWSUDs.

**Electronic supplementary material:**

The online version of this article (10.1186/s13011-018-0185-y) contains supplementary material, which is available to authorized users.

## Background

Substance use and substance use disorders (SUDs) are clearly and consistently associated with an increased risk of suicide, which is a major public health concern, particularly in low- and middle-income countries (LMICs) [1–4]. It is estimated that globally 800 000 people die by suicide annually, which has led to calls by the World Health Organization (WHO) for countries to develop national suicide prevention strategies and deliver interventions targeted at high-risk groups who are vulnerable to suicide [2]. Targeting high-risk groups has been shown to lead to reductions in suicide rates [3–5]. Although people with substance use disorders (PWSUDs) are a clearly delineated group who are at elevated risk of engaging in fatal and non-fatal suicidal behaviour [6, 7], it is not always clear what strategies should be employed to curb suicidal behaviour in this group, particularly in countries like South Africa (SA), where mental health resources are scarce, health care is often inaccessible, and services for PWSUDs are fragmented and unintegrated [8]. In SA there is increasing recognition that generic strategies for health care provision need to be augmented or replaced by strategies tailored towards the specific needs of particular groups in order to reduce health inequalities [9]. Similarly, suicide prevention efforts need to target vulnerable and high-risk populations, like PWSUDs, and interventions need to be tailored to particular socio-cultural and economic contexts [10, 11]*.* While mental illnesses are associated with suicide [12], broader socioeconomic factors (such as poverty and cultural norms) also play an important role in suicidal behaviour [13–15]. Mental health care providers (MHCPs) come into regular contact with suicidal individuals and are tasked with preventing suicide [16–19]. As such, they may have valuable insights into (a) what may be required to prevent suicide among PWSUDs and (b) the particular social, cultural, economic, political, or health care contexts that need to be considered when planning suicide prevention interventions for PWSUDs. These insights may be used to inform specific suicide prevention strategies that are sensitive to the particular needs of PWSUDs, offering greater specificity than generic intervention strategies such as the World Health Organization (WHO) guidelines on suicide prevention [2]. It is within this context that we set out to explore MHCPs' suggestions for preventing suicide in PWSUDs in SA.

### Substance use in SA

SUDs are as prevalent in SA as in other regions of the world [20, 21]. The South African Stress and Health Study, a population-based study of common mental disorders in SA, found that the lifetime prevalence of substance use disorders is the second highest among classes of common mental disorder at 13.3%, with alcohol abuse as the most common single mental disorder at 11.4% [22]. Further data from this study show that alcohol is the most used substance in the SA population (38.7%), followed by tobacco (30%), cannabis (8.4%), and other drugs (2%), although this last statistic was thought to be an underestimate [23]. Regarding more recent data, the South African Community Epidemiology Project on Drug Use (SACENDU) project is a sentinel surveillance system in SA that collects data from rehabilitation centres across the country regarding prevalence rates for alcohol and other drugs with the exception of tobacco. Patterns of substance use in SA differ by region, and the latest SACENDU data from July to December 2017 shows that clients in the Western Cape of SA present for treatment for multiple substances, with methamphetamine being the most common (30%), followed by alcohol (24%), cannabis (22%), heroin (14%), and lastly a variety of other drugs [24]. The Western Cape shows the highest rates of SUDs in the country [22].

Treatment for mental health problems in SA is provided within two parallel health care systems – a publically-funded system which serves the majority of the population and a private health care system that provides services to the country’s wealthy, mostly white, minority. The public health care system is characterized as overburdened and under-resourced [8]. Treatment for SUDs in SA is overseen by the Department of Social Development, with input from the Department of Health mostly for management of detoxification from substance use disorders and treatment of co morbidities [25]. There are a limited number of state-run substance rehabilitation services with a limited number of available beds in hospitals for treatment of SUDs. In recent years, multiple efforts have been made to address this gap including the provision of evidence-based Matrix Model outpatient treatment centres at primary health care clinics within the City of Cape Town [26, 27]. There are also a number of private substance treatment facilities and services provided by non-profit organizations, including churches. These facilities are typically staffed by a wide variety of service providers (including psychiatrists, psychologists, social workers, professional counsellors and lay counsellors). Demand for SUD treatment services in SA far exceeds supply, and barriers such as cost of treatment, access to transport, not knowing where to access services, burdensome travel distances, and more pressing financial priorities combine to prohibit PWSUDs from accessing care [28–30].

### Suicidal behaviour in SA

Suicide is a serious public health concern in SA, with official statistics suggesting that the prevalence of suicide is 14 per 100 000 [31]. The South African Stress and Health Study estimated lifetime prevalence of suicidal ideation, suicidal plans and suicidal attempts at 9.1%, 3.8%, and 2.9% respectively [32]. There is a growing body of literature from SA showing that rates of substance use are high among individuals who complete suicide [31] and individuals who engage in non-fatal suicidal behaviour [33, 34]. Suicidal individuals can receive mental health services from public health facilities (clinics, regional hospitals, and academic hospitals), MHCPs working in private practice, or free suicide helplines (such as those provided by LifeLine and SADAG). While there are comparatively more mental health services in the Western Cape compared to other provinces [35], the majority of MCHPs work in private practice and their services are therefore not accessible to the majority of the country’s population [36, 37].

### Suicide prevention strategies

There is a large body of literature describing suicide prevention strategies. The WHO suggests generic strategies at three levels (see Table 1 for a summary) while various governments have proposed national suicide prevention strategies that combine broad directions and more specific suggestions for suicide prevention [38–40]. Additionally, professional organisations have proposed standard treatment guidelines and clinical practice guidelines, which provide specific direction to clinicians on best practice in the assessment and management of suicidal behaviour [41–46]. Evidence-based interventions such as Collaborative Assessment and Management of Suicidality (CAMS) [47–50] and The Safe Alternatives for Teens & Youths Program (SAFETY) [51] describe very specific interventions for preventing suicide in high-risk individuals. Tables describing examples of governmental suicide prevention strategies, guidelines proposed by professional organisations, and specific suicide prevention strategies can be found in Additional File 1.

**Table 1:** Levels of suicide prevention proposed by WHO [2]

Level of approach	Description
Universal	Target the general population and focus on health promotion, through strategies such as: reducing access to means; improving access to mental health care; and reducing harmful substance use.
Selective	Target vulnerable groups through strategies such as training gatekeepers to identify warning signs and establishing services such as helplines.
Indicated	Target specific high-risk individuals, like PWSUDs, by improving access to mental health care, improving identification and management of suicidal behaviour, offering community support, and increasing training and education for health care professionals working with suicidal patients.

Systematic reviews show that means restriction, improving access to mental health care, improving the abilities of health care providers (especially general physicians) to screen for and manage depression and suicide risk, and training community gatekeepers to recognise suicide warning signs and link at-risk individuals with mental health services are all effective ways to prevent suicide [52–54]. These strategies are typically implemented as isolated or stand-alone interventions and therefore attend to only a single risk factor for suicide. To address this, national suicide prevention strategies have been designed as integrated, multi-level social ecological approaches to suicide prevention [38–40]. There is evidence to show that such multi-level strategies, which employ universal, selective and indicated interventions simultaneously, produce greater reductions in suicide than isolated interventions [55]. For these interventions to be effective, intersectoral collaboration and integrated health care are required.

Critical suicidologists have argued that dominant approaches to suicide prevention have had limited effectiveness because they focus too narrowly on individualist, biomedical and psychiatric approaches to suicide prevention and do not account for context- and population-specific factors [15]. To advance research and practice in suicide prevention, the cultural, social, economic, and political antecedents and contexts of suicidal behaviour need to be considered [10, 11, 15, 56].

### Suicide prevention in SA

In SA, progress has been made in the past 20 years to prioritise the mental health needs of the population and improve mental health services in the country. However, a lack of intersectoral collaboration and integrated care have been noted as barriers to suicide prevention—particularly among PWSUDs [13]—and the provision of mental health care in general [57]. Despite the agenda for research on suicide and its prevention suggested by Bantjes and Kagee [31] and the possible suicide prevention strategies and plans suggested by Burrows and Schlebusch [58] and Schlebusch [56], no progress has yet been made to plan or implement coordinated, targeted suicide prevention interventions in SA. The National Mental Health Policy Framework and Strategic Plan 2013-2020 mentions the need to develop a national suicide prevention strategy and embed suicide prevention at a primary care level [59], but no national governmental guidelines stipulating how suicide should be prevented in the local context have been promulgated. This may be due to prioritisation of other health needs and a lack of research on what specifically is required to prevent suicide, particularly amongst high-risk populations.

Research in SA and other low- and middle-income countries (LMICs) has indicated that there are specific contextual variables that need to be accounted for when preventing suicide in PWSUDs [13, 60]. The concentration of PWSUDs in the Western Cape and the clear link between SUDs and suicide [6, 7] means that understanding what is needed to prevent suicide amongst this high-risk group has immediate clinical relevance to MHCPs. Understanding what is required for suicide prevention amongst high risk-groups, such as PWSUDs, may help contribute to the development of a national strategy for suicide prevention.

## Methods

### Aim, design and setting

Our aim was to explore MHCPs' suggestions for preventing suicide among PWSUDs in the Western Cape, SA. The qualitative design of this study allowed for exploratory investigation of an under-researched topic of clinical relevance to MHCPs, particularly those working with PWSUDs in LMICs. The setting of this study included the public, private and academic sectors of the SA health care service as well as substance use treatment services, which are under the management of the Department of Social Development. These services are adversely affected by fragmentation in service provision and a lack of staff and other resources [8, 13].

### Sampling and participants

Participants were recruited using purposive and snowball sampling until a point of data saturation was reached. JB and LD have worked in the SA mental health care setting for over a decade each and were able to suggest a limited number of potential participants during the purposive sampling phase who had experience working with suicidal PWSUDs. In total, 21 potential participants were approached to participate; three did not respond to invitations to participate and 18 consented to be interviewed. In recruiting these participants we employed the concept of “maximum variation sampling” by deliberately including participants from a wide and varied range of settings and a variety of disciplines, in order to maximize the diversity of experiences and insights. Ten participants were recruited using purposive sampling (four psychiatrists, four clinical psychologists, two counsellors) and eight participants (four social workers and four counsellors) were recruited using snowball sampling.

### Data collection

DG conducted in-depth, semi-structured interviews in English with all participants. Each interview was conducted at a time and location of the participant's choice. Interviews ranged between 40 and 90 minutes. Participants were asked about their ideas for preventing suicide in PWSUDs, with a focus on what they thought was effective in their work as well as what is needed in the SA context. Participants were asked to elaborate and clarify their ideas for suicide prevention when they appeared to be nonspecific by asking questions such as "Could you provide more detail about that suggestion?" and "What specifically do you mean by that?". All interviews were digitally audio-recorded and transcribed. Data were collected between 02 September and 30 November 2016.

### Data analysis

The data were analysed using thematic analysis [61]. Initially, DG coded the data inductively, and JB reviewed the coding in an effort to improve the rigour of the research by verifying the coding independently. Once codes had been agreed on, DG and JB grouped codes into themes that captured the underlying data, resulting in nine subordinate themes that were grouped into three superordinate themes (providing effective mental health care, mental health care system reforms, and community intervention and proactive prevention). Throughout data collection and analysis, detailed field notes and process notes were made in order to promote dependability [62]. All authors reviewed the themes to ensure that they reflected the interview content, to improve the confirmability of the findings [62]. LD conducted a confirmability audit to ensure that all interpretations were supported by corresponding data [62]. Quotes are included to provide evidence for the findings and to enhance credibility, and all participants are anonymised by referring to them according to their professional identities. Data from all 18 interviews are included in the findings to prevent bias towards/against any data. The data were analysed using the software program Atlas.ti. The data presented here form a subset of the total findings. This article focuses on themes relating to MHCPs' ideas for preventing suicide in PWSUDs. Other findings, published elsewhere, describe the experiences of MHCPs in preventing suicide in PWSUDs [63] and the barriers they perceived to preventing suicide in PWSUDs [13]. These findings were described in three separate articles in order to allow for thorough description and substantial discussion of the findings.

## Results

### Sample characteristics

Participants had a collective 173 years of experience working with suicidal PWSUDs and the median number of years worked with suicidal PWSUDs was six years. Participants had experience working in a wide range of settings, including emergency, outpatient and inpatient psychiatric settings; private and public outpatient and inpatient SUD treatment facilities; private practice; community health services; NGOs; schools; universities; and government departments. At the time of data collection, three participants were working in psychiatry departments within academic hospitals providing services to psychiatric inpatients and outpatients, three were working in private practice providing psychiatric and psychotherapeutic substance use treatment services, nine were working in outpatient state-funded substance use treatment facilities providing counselling and social work services to clients with a primary diagnosis of a SUD, two were working in NGOs providing counselling services to PWSUDs, and one was a clinician with experience of providing treatment to PWSUDS who was currently completing a doctoral degree in the field of substance use.

### Providing effective mental health care

#### Instillation of hope

MHCPs said that they "*have to provide hope*" and empower PWSUDs in order to protect them from suicide. A social worker stated, "*there is always value in a person, but they don’t always see it*." A clinical psychologist noted the importance of helping PWSUDs find a "*sense of purpose*" by helping create "*future goals*." Providing hope was said to protect PWSUDs against suicidal behaviour and help motivate them to address their substance use. Some suggested that providing PWSUDs with employment opportunities and homes would be a way to engender hope. Participants affirmed that in order to help their clients reconnect to hope, it was necessary for them to remain optimistic and believe in the autonomy and capacity of PWSUDs to heal themselves and make positive changes in their lives. They said they achieved this by adopting a humanistic stance and strength-based approach to interventions:

*[A]s a mental health professional, you want to kind of seed hope, and I’m always kind of looking for like a strengths-based approach and I guess like 'let’s see what you have and how we can build on that.'*

#### Establishing a therapeutic alliance by focusing on the relationship

Participants affirmed the importance of building “*a trusting relationship*” with suicidal PWSUDs by communicating “*compassion*” and “*kindness*.” One participant explicitly advocated for the effectiveness of a "*Rogerian approach*" by maintaining “*unconditional positive regard and acceptance*”. This sentiment was echoed by another participant who said:

*I think a client would need just, at that stage to be listened to completely and putting every other therapeutic goal, just, away, and just being there with that client at that stage. I think that in itself... will be effective*.

Participants affirmed the importance of authenticity (being "*empathic and to be real, to be real in the relationship*"), establishing "*rapport*", and maintaining "*a focus on the relationship*". One participant said that the experience of an authentic relationship characterised by empathy provided "*a different experience to what they get in a community*." Another participants said that it was important to provide containment as an antidote to suicide, by being somebody who could "unders*tand... set boundaries, [and] think through what’s going on.*"

### Effective treatment for psychiatric conditions

Participants said that effective treatment of SUDs and other psychiatric conditions would significantly decrease suicidality. This was evident in the words of one participant who said: "*treating the addiction... will definitely help reduce the suicide risk*". In this context participants suggested a "*step-down*" approach to treatment and highlighted the importance of post-discharge and on-going out-patient care. Participants also advocated for a "*harm reduction*" approach to treatment of SUDs in order to prevent suicide. They also suggested that "access to an immediate response of some sort" was critical when PWSUDs were in a suicidal crisis. Participants highlighted a need for MHCPs to be trained in evidence-based approaches to both the treatment of SUDs and suicidal behaviour. They said that this needs to be based on a model that allows patients to manage "*triggers in their environment*" that make them want to use substances or make them feel suicidal.

Participants noted how important it is to accurately assess and treat comorbid mental health conditions, especially depression, in order to prevent suicide. A clinical psychologist affirmed the importance of doing “*a systematic inquiry*” to “*exclude major depressive disorder or an anxiety disorder,”* and of providing effective treatment for these conditions when assessing and managing PWSUDs.

*Obviously one of the big areas is dual diagnosis—patients with a substance use disorder and another mental health problem—and for those patients, access to mental health services and specialist mental health services is actually very very important... you can’t treat the one without the other.*

### Mental health care system reforms

#### Training and the establishment of minimum norms and standards

All participants noted a need for better training for all health care providers in suicide prevention. They said MHCPs need to be equipped to conduct thorough risk assessments when working with suicidal patients, and that they needed to receive training in brief evidence-based interventions such as the use of motivational interviewing. Participants said there was a need to include more suicide-specific training for MHCPs to *"prepare them in dealing with suicidal patients. Identification, features and that. I think we can do a lot more work in training with that."*

Participants said that in order to provide effective interventions, there should be minimum norms and standards for the training and accreditation of MHCPs who work with PWSUDs. They also suggested that treatment facilities be better monitored to ensure that they are registered with the correct government department.

*There should be some kind of minimum requirements to say if you want to work in this area, you know, you need to have at least a diploma in addictions or something like that, a postgraduate diploma. But I’m not sure if it’s regulated in that way.*

#### Provide integrated and comprehensive care

Participants noted a need for more integrated service provision and intersectoral collaboration, where different health care providers, MHCPs, community/non-governmental organisations, and government departments (i.e., the Departments of Health and Social Development) work in harmony to prevent suicide, treat mental health problems and address problematic substance use. Advocating for the importance of multi-disciplinary teams, one participant said:

*[W]here most of the difference is actually gonna be made in an ideal world is post-acute in the sort of recovery phase, you know, so to be able to have not only regular, sort of, frequent consultations with allied mental healthcare workers, we need to have like a multi-disciplinary team that is managing these people as out-patients, so in the ward, you’ve got a social worker, an OT, a psychologist, a psychiatrist, and all of that—why does that end when you leave? You know? You need to have the same inputs.*

A psychiatrist said that MHCPs need to intervene beyond addiction as "*you treat the addiction, but it remains hopeless... if you can’t help the patient rebuild their life*." A counsellor noted that "*the resource that we lack the most is connectivity and integration... systems integration*." Using multi-disciplinary teams to provide "*integrated treatment*" for SUDs and mental health conditions was also said to be integral to suicide prevention. Participants noted that this allows "*input from different experts who focus on different things*" meaning that PWSUDs could "*work through those different elements that are contributing to suicidality*." . Participants suggested linking multiple types of services to provide integrated care:

*I think just more, a more coherent system, where there's sort of interlinked services, between health, between social services, where it's all much more interlinked, but I think also linked up again with things like self-development, job creation opportunities, housing opportunities, so that it becomes more community orientated, and again I'm not negating the medical, because I do think that the medical is very important, and again, often with substance use there are comorbid physical conditions, and I think those can't be ignored either. So I really think sort-of, one needs a coherent approach, where the physical, the medical's looked at, medication's looked at, psychiatric, as well as psychological relationships, social stuff, ideally.*

#### Improve availability and utilisation of resources

When it came to providing integrated and comprehensive care to prevent suicide, participants expressed plainly that "*we just need more resources to be able to do these things."* Resources included financial support, infrastructure ("*more beds*"), health services (including "*community services*", "*clinics to deal specifically with addiction*", "*halfway houses*"), and personnel ("*social workers*", "*nurses*", "*psychologists*", "*occupational therapists*"). More resources would allow more specialised service provision for patients with SUDs and/or suicidal behaviour. One participant articulated this by saying:

*Good quality acute care [is needed to help] effectively manage the risk and help that person get to a position where they're more able to engage, and develop coping skills and strategies around that*.

Some participants expressed a need for resources such as a "*suicide safety room"* to deal with a suicide crisis "*on site*." Having more resources was suggested as a way to relinquish some of the burden on existing MHCPs, allowing them to make "*a bit more of a mind shift*" and give "*a bit more time*" and thought to suicidal patients. While participants said that more resources are undoubtedly needed, they also said that existing resources might be better utilised.

*[T]here are so many substance abuse centers in Cape Town, but nobody knows, where they are or how to get into contact with us or how to go about so, I think even with, with suicide, a lot of people don’t know where to turn to or who to, who to talk to about it.... There’s counselors [sic] available, there’s helplines [sic] available, there’s groups [sic] that is running [sic] on a day-to-day basis, there’s psychiatrists [sic] at hospitals, it’s a phone call away. There’s hospitals that is 24/7 [sic], you know, so it’s not like there’s no help. There’s hands [sic] waiting to grab onto whoever’s sticking their hand out.*

Participants said that they need to optimise referral pathways, "*redistribute resources more cleverly*" and "*think creatively around*" resource allocation to prevent suicide. Participants suggested that "*task shifting*" and "*supervision*" be utilised as an effective and cost-effective way to decrease the burden on more highly-trained MHCPs (such as psychologists and psychiatrists). Referring to hospitals and SUD rehabilitation facilities, a counsellor suggested that "*if they can’t afford clinical psychologists, they can employ registered counsellors*." This perception was echoed by a participant who said:

*I think there’s an opportunity to train, you know, kind of, a tiered system… people that are coming out with an undergraduate degree who could then go off and do an extra year internship or a placement like you know, kind of somewhere, get- and in that placement do some kind of counselling diploma, they get registered as a counsellor. I mean the structure’s there. [The idea is that] they get trained in suicidality, they get trained in substance abuse, they get trained in a whole lot of different areas. But the rules of their registration are that they have to be in supervision.*

### Community interventions and proactive prevention

#### Provision of emotional and social support

MHCPs said that providing support (from family, friends, and other suicidal individuals) should be a critical component of suicide prevention. Participants suggested improving community cohesion and said that family and friends were important sources of support. A psychiatrist noted that PWSUDs need "*aftercare and support*," while others said that providing support would make patients feel less alone and more prepared to deal with a suicide crisis themselves. A “*suicide support group*” similar to Narcotics/Alcoholics Anonymous was suggested as a place where people who felt suicidal, or who had experienced or engaged in suicidal behaviour in the past, could support one another.

#### Interventions to prevent substance use and mental health issues

Participants mentioned the importance of good childcare practices, primary school education, and interventions such as the "*good behaviour game*" to act as a prophylaxis against adversity. Having loving parents and strong communities were mentioned as important ways to prevent later substance use and suicidal behaviour, as a clinical psychologist said that "*if every child had a caregiver who truly, who truly cared for him or her, that in itself I think society would change radically*." Teaching "*coping skills*," stress tolerance, general "*life skills*," interpersonal skills, and "*communication skills*" to youths was highlighted as important to help promote mental health, prevent suicidal behaviour and "*change the trajectory of these kids*."

Creating awareness in communities around how to deal with suicide, how to identify the warning signs of suicide, what resources are available, and to improve people's understanding of SUDs and suicidal behaviour were suggested to improve help-seeking behaviours. Public education initiatives and pamphlets in doctors' waiting rooms were suggested in addition to psychoeducation in schools. Social media was suggested as a platform to disseminate information about mental health, substance use, suicidal behaviour, and help-seeking, through creating government-funded websites that can be accessed free of charge.

*I think people should be more observant of the signs. So I think people in general should be educated about what to look out for cos like now we say the people don’t really want to share the, the—“I’m now suicidal”. So I think, communities, families, there needs to be more to say “this is kind of stuff you need to look out for”. Teenagers, I mean if I look at our clients, most of them started already experiencing it in their teenage years, cos it was then already that they couldn’t deal with certain things.*

#### Reduce stigma about substance use and suicidal behaviour

Many MHCPs said that a critical part of preventing suicide was to demystify and destigmatise substance use and suicidal behaviour. Participants said that "*we've gotta have this rational conversation around drugs*" and "*we need to talk more about suicide*" in order "*to get rid of the stigma*." Participants said that this is likely to improve treatment adherence, acceptance of the fact that one needs help, support from loved ones, and help-seeking behaviour. Participants suggested that stigma be reduced amongst medical staff, MHCPs, and the general public. As such, stigma-reduction initiatives could be a part of public education campaigns and training of MHCPs, and were suggested as imperative to suicide prevention initiatives.

*We need to do something about stigma. It’s very—it’s still very prominent here, uhm the, "the drug addict decides to be a drug addict" or the suicidal person who’s "selfish" cos they, you know wanna kill themselves, but at the same time we don’t actually offer any kind of treatment in most places in the country. Uhm and the—or the, the families are very harsh on, on people who have got substance use problems, or they’re in denial.*

## Discussion

In the context of making suggestions for suicide prevention among PWSUDs, participants in this study spoke about the importance of providing emotional and social support as part of comprehensive mental health care interventions that entail the instillation of hope in service users. They affirm the importance of providing effective treatment for psychiatric conditions within an integrated health care system. They argue for increasing the availability of resources for SUD treatment and suggest how resources might be utilized differently to improve the standard of care provided to PWSUDs. They suggest a restructuring of the mental health care system and better training of existing MHCPs. Furthermore, participants assert the importance of community-based interventions focused on early detection and prevention of substance use and mental health problems, and reducing stigma. Many of these suggestions are supported by the suicide prevention literature [52–54]. It is remarkable that much of what is suggested by the participants in our study is not specific to suicide prevention and simply reflects principles of good mental health care provision. It is not immediately clear what to make of this lack of specificity in participants’ suggestions for suicide prevention among PWSUDs, but there are at least four possibilities which we discuss below.

First, the lack of specificity in the suggestions made by our participants may simply reflect participants' beliefs that much of what health professionals do when they attempt to prevent suicide amongst PWSUDs specifically, is not inherently different from what they do when they are providing good mental health care services more generally. Establishing rapport, listening with empathy, instilling hope, providing containment, and helping the patient develop their own internal and social resources are key elements of a person-centred approach to mental health care and form the basis of many psychological interventions. The implication of this is that general principles of effective mental health care provision are the cornerstone of suicide prevention amongst PWSUDs. This perception stands in contrast to much of the suicide prevention literature which constructs suicide prevention interventions as something fundamentally different from routine psychological care. There are a number of theoretically-underpinned, evidence-based suicide prevention interventions, including CAMS [47–50], SAFETY [51], and cognitive-based therapy for suicide prevention [3, 64]. The tension between generic, transdiagnostic approaches and disorder-specific approaches to suicide prevention echoes similar debates concerning the treatment of anxiety disorders, eating disorders, and SUDs [65–68]. Training MHCPs in the fundamental principles of empathic, person-centred care is important, but there is a clear need to move beyond generic approaches to draw on the existing evidence base of suicide prevention interventions.

A second possibility is that the lack of suicide-specific suggestions made by our participants reflects their lack of training in suicide prevention and, more importantly, their lack of awareness of evidence-based interventions or contemporary theories of suicide. This is concerning, given the centrality of suicide prevention in their work treating substance use disorders [13, 63]. The implication of what our participants say is that there is an urgent need for training to enhance the competence of MHCPs working with PWSUDs to deliver evidence-based suicide prevention interventions. In this context, it is noteworthy that our participants spoke of a perceived lack of legislation and minimum norms and standards to oversee the training, qualifications, and competencies of those who provide treatment to PWSUDs in SA. The existing minimum norms and standards for SUD treatment facilities in SA do not specify the exact training required by employees of such treatment facilities and do not require a MHCP registered with the Health Professions Council of South Africa to be a part of the treatment team [69, 70]. Indeed, not all treatment centres in SA are registered with the Department of Social Development and not all treatment centres employ adequately qualified staff [71]. Core competencies are specified in the policies outlining minimum norms and standards for SUD treatment centres, but do not include the ability to treat comorbid mental health conditions or, critically, to prevent suicide. There is also no specification for how a multi-disciplinary team might be utilised, and these participants provide some helpful suggestions in this regard. Some of the problems associated with these norms and standards have been noted by other scholars [72, 73] and need to be addressed to help prevent suicide amongst PWSUDs. There is an obvious need to revise such policies to specify that accredited, adequately trained MHCPs are employed in SUD treatment facilities. Training in evidence-based suicide prevention interventions could be added to the minimum norms and standards as a core competency for those working in SUD treatment facilities or for those working with PWSUDs more generally. This relates to a more general need for greater specificity regarding the core competencies required by MHCPs in SA. While progress has been made to transform the provision of mental health care in SA through, for example, the promulgation of the National Mental Health Policy Framework and Strategic Plan 2013-2020 [59], core competencies are poorly specified in existing policies [74]. Many of these participants' suggestions could provide direction for the improvement of policies governing the treatment and prevention of SUDs and mental health problems in SA.

A third possibility is that participants underestimated the specialised nature of providing mental health care to suicidal PWSUDs. Participants suggested shifting the responsibility for (a) preventing suicide, (b) treating SUDs, and (c) treating comorbid psychiatric conditions to registered counsellors, who only have four years of basic training in psychology and counselling (compared to clinical psychologists — at least six years of training, including two years of intensive supervised practice, and psychiatrists — at least 10 years of training, including four years of intensive supervised practice). Their suggestions to utilise task-shifting are understandable given some success of similar interventions in SA [75]. However, substance use is a specialised field in psychiatry and clinical psychology and preventing suicide requires specialist training under close supervision. Expecting MHCPs trained in basic counselling skills to be able to competently manage and treat the complexity of suicide, SUDs, and comorbid psychiatric disorders may be overly optimistic. While their suggestion is indicative of a need to think creatively around mental health care provision and utilise all cadres of MHCPs, employing more MHCPs requires job creation and funding that is not available in the public sector in SA. Training non-professional counsellors in specific suicide prevention interventions may be effective in reducing suicidal behaviour [76, 77] but reductions in suicidal ideation and attempts appear to be better when a mental health care professional administers the intervention [7]. More research is required to assess the feasibility, efficacy and sustainability of implementing task-shifting to help prevent suicide among PWSUDs.

The fourth possibility is that, in the suggestions they make, our participants are reproducing a dominant contemporary discourse about mental health care in SA. They talk about a need to address: inadequate mental health care, poor access to psychosocial services, a marked lack of integrated person-centred care, resource constraints, and an absence of social support. They advocate for better services, more MHCPs, task-shifting, and community-based substance use and suicide prevention programs. Combined efforts by multiple sectors would help support suicide prevention initiatives in the health sector, but challenges to intersectoral collaboration have been noted previously [57]. Our participants' ideas are important and have the potential to help radically transform the mental health care climate in SA. However, they are not novel suggestions and they were at the heart of the mental health advocacy zeitgeist in SA during the time we collected our data. In this context, it is worth noting that we collected data at the time that news about the Life Esidimeni tragedy was breaking. Briefly, mentally ill patients were transferred at the direction of the regional Department of Health from the Life Esidimeni psychiatric hospital to unlicensed NGOs [78, 79]. Over 140 patients died during this process due to negligence, starvation, and inadequate care provision [78, 79]. This tragedy highlighted the inadequacies in mental health care provision in the country, how decreased funding and poor decision making result in adverse health outcomes (including death) among psychiatric patients, the obvious need to transform mental health care provision in SA, and the consequences that befall the marginalised mentally ill when mental health is not prioritised by the government.

In reproducing dominant discourses about mental health care crises in SA, our participants remind us that the treatment of PWSUDs and efforts to prevent suicide in this population occur within a broader political, economic, and mental health care context. Addressing economic, political, and mental health care factors in order to help prevent suicide and SUDs is as important as intervening on an individual level, and may be more important in SA. Such a perspective offers a radical departure from traditional early intervention strategies for suicide prevention that continue to emphasise raising awareness about suicidal behaviour and improving access to health services [80]. Redistribution of resources, improving childhood mental health, and transforming the economic and health care contexts of SA must occur in parallel with programmes directed at equipping existing MHCPs with more specialised training in suicide prevention.

### Limitations

This study was conducted in the Western Cape, SA, which has some of the highest rates of mental health problems in the country [21]. As such, these participants may have provided services for a population with particularly severe SUDs, and may also see more psychiatric comorbidity and social issues. This means that these findings may not be applicable in other regions of SA or in other LMICs. Limitations of space necessitated omission of some longer quotes in the findings. This may detract from the perceived confirmability of the findings. Despite these limitations, this study provides a useful first step in suggesting how suicide prevention interventions can be tailored to the specific needs of PWSUDs.

## Conclusions

Suicide prevention, particularly among PWSUDs, is challenging. Although there do not appear to be simple solutions to the problem of how to reduce suicide risk, there is a growing evidence base of promising strategies which MHCPs can draw on in their work to prevent suicide among PWSUDs. The suggestions for suicide prevention made by the participants in this study remind us that the work of suicide prevention, particularly among PWSUDs, can only occur within the context of large systemic changes in the delivery of mental health care and treatment for SUDs in SA. Training MHCPs to deliver evidence-based suicide prevention interventions while failing to address larger systemic problems in the delivery of health care is likely to be ineffective. Rethinking how we conceptualise and deliver suicide prevention interventions may require broadening our frames of reference beyond individualist, biomedical perspectives to include a focus on economic, political, and socio-cultural environments that influence suicide and its prevention, especially amongst PWSUDs.

## Additional file

Additional file 1: Tables describing examples of governmental suicide prevention strategies, guidelines proposed by professional organisations, and specific suicide prevention strategies. (DOCX 32 kb)

## Abbreviations

LMICs: Low- and middle-income countries
MHCP: Mental health care provider
NGO: Non-governmental organisation
PWSUD: Person with a substance use disorder
SA: South Africa
SUD: Substance use disorder
WHO: World Health Organization
