# Supplementary material for: Mental health care providers' suggestions for suicide prevention among people with substance use disorders in South Africa: a qualitative study
Source: Subst Abuse Treat Prev Policy. 2018 Dec 7;13:47. doi: 10.1186/s13011-018-0185-y (PMC6286590; doi:10.1186/s13011-018-0185-y)
Supplement: Supplementary file 1 — Tables describing examples of governmental suicide prevention strategies, guidelines proposed by professional organisations, and specific suicide prevention strategies. (DOCX 32 kb) [file 13011_2018_185_MOESM1_ESM.docx]

Table S1: Examples of national suicide prevention strategies.

| **Country** | **Guiding principles** | **Action areas** |
| --- | --- | --- |
| United States [1] | - Healthy public dialogue about suicide should be fostered - The needs of vulnerable groups must be prioritised - Interventions should be applied in a context-specific manner - Interventions should be coordinated and integrated - Changes in policies, systems and environments to prevent suicide should be promoted | - Build healthy and empowered individuals, families, and communities, by integrating suicide prevention interventions across sectors and settings, changing negative attitudes and behaviour, increasing knowledge of protective and wellness-related factors, and promoting responsible media reporting of suicide - Provide clinical and community preventive services, including implementing effective programmes to promote wellness and prevent suicide, reducing access to means, and training service providers to prevent suicide - Provide treatment and support services, by making suicide prevention a core component of health services, implementing effective suicide risk management protocols, and providing care and support to suicidal individuals - Strengthen research on suicide prevention |
| Australia [2] | - Suicide prevention activities will do no harm - There will be community ownership and responsibility for action to prevent suicide - Service delivery will be client-centred - The responsibility for suicide prevention lies with individuals, professional groups and services across the community - Interventions should be provided in a coordinated and integrated way according to the needs of the individual and community - Suicide prevention activities will occur across eight overlapping domains of care and support - Safety nets should be provided to support people moving between treatment options, and back into the community | - Improve the evidence base and understanding of suicide prevention. - Build individual resilience and the capacity for self-help - Improve community strength, resilience and capacity in suicide prevention - Take a coordinated approach to suicide prevention - Provide targeted suicide prevention activities - Implement standards and quality in suicide prevention |
| Japan [3] | - Comprehensive approaches take social factors into account - Efforts should be made to have individual citizens play a leading role in suicide prevention - Measures should be combined effectively to deal with each stage and each target group - Inclusive support must be strengthened for everyday life by coordinating all those concerned - Policies must be based on actual conditions of suicide - Policies are tested and evaluated from a mid- and long-term perspective - Suicide prevention measures are promoted based on the actual conditions of each targeted group - Efforts between government, NGOs, local authorities, businesses, related organizations, and the general public must be coordinated to have maximum effects on suicide prevention | - Clarify the actual conditions of suicide - Encourage citizens to be aware of and monitor potential suicide victims - Train personnel who play a central role in early response - Promote mental health - Ensure that appropriate psychiatric care is received - Prevent suicide through social measures - Promote measures to deal with suicide-related information on the internet - Prevent repeat suicide attempts - Improve support for the bereaved - Strengthen coordination with NGOs |

Table 2: Examples of suicide prevention clinical practice guidelines and the strategies they advocate.

| **Professional bodies and governmental departments that have proposed clinical practice guidelines for suicide prevention** | **Underlying principles** | **Strategies for suicide prevention** |
| --- | --- | --- |
| National Institute for Health and Care Excellence in the United Kingdom [4-6] | - Treat patients with compassion, respect and dignity - Adopt a non-judgemental approach to the patient - Ensure that patients are consulted in the treatment process - Ensure health care providers are adequately trained to manage risk of suicidal behaviour - Health care providers work cooperatively while caring for suicidal individuals | - Acute-care interventions, including treatment of self-inflicted injuries, acute care management, and risk assessment - Treatment of underlying psychopathology - Referral to specialised services - Activation of psychosocial support - Designing long-term care and risk management plans |
| American Psychiatric Association (APA) [7] | - Thorough psychiatric evaluation is imperative for managing suicide risk - Respect for cultural differences - Health care providers work cooperatively while caring for suicidal individuals | - Psychiatric evaluation, including assessment of suicide risk - Establish a psychiatric management plan, including treatment of psychopathology with pharmacological and psychotherapeutic means - Risk management - Psychoeducation for family of suicidal individual - Strengthen research on suicide prevention |
| Spanish Ministry Of Health, Social Services And Equality [8] | - Suicidal behaviour should be addressed from a broad perspective - Comprehensive assessment of suicidal individuals should involve health professionals from different levels of care - The development of a strong therapeutic alliance between patient and professional should be promoted - Establishing support in the patient environment is a fundamental part of the therapeutic process - Patient is consulted at all stages of treatment | - Acute medical treatment of suicidal behaviour-related injuries - Comprehensive psychiatric assessment, including assessment of suicide risk and associated risk factors - Refer high-risk patients to specialised services - Treat suicidal behaviour and psychopathology with pharmacological and psychotherapeutic means |

Table 3: Specific, evidence-based suicide prevention protocols and their key features.

| **Protocol** | **Underlying principles** | **Key treatment features** | **Examples of studies showing evidence for effectiveness** |
| --- | --- | --- | --- |
| Collaborative Assessment and Management of Suicidality (CAMS) [9] | - Empathy for the suicidal individual - Collaboration between clinician and patient regarding suicide assessment and risk management plan - Honesty and forthrightness with the patient - Maintaining clinical documentation that reflects excellent practice helps decrease liability - Flexibility and adaptability across theoretical orientation, discipline, and clinical setting is central. | - Establish a strong clinical alliance and increase patient motivation - Thoroughly and comprehensively assess suicidal risk. - Develop and maintain a problem-focused treatment plan that is suicide-specific - Sit next-to, rather than across from, the suicidal individual when working out a risk management plan - Use the Suicide Status Form to guide assessments, treatment planning, tracking of ongoing risk, and clinical outcomes - Focus is only on suicide prevention, not on treatment of comorbid conditions | [10-13] |
| The Safe Alternatives for Teens & Youths  (SAFETY) Program [14] | - Rooted in a social-ecological cognitive-behavioural model - Focus specifically on preventing suicidal behaviour in adolescent suicide attempters - Emphasise enhancing protective supports within social systems (family, peers, community) - Family focus aims to increase parent motivation and decrease family treatment barriers - Treatment is guided by a cognitive-behavioral fit analysis (CBFA) that specifies key risk and protective processes hypothesised to impact the likelihood of repeat suicide attempts for each youth. | - CBFA of suicidal behavior; including explaining how suicidal behavior ‘‘fits’’ within multiple systems (families, peers, school, community) through an ecological analysis, identifying risk and protective factors to be targeted through treatment plan - Enhance protective familial support and monitoring in the home - Ensure a safe environment by restricting access to potentially lethal methods - Develop and practice SAFETY plan, to change maladaptive thought patterns - Collaborate with youth and family to develop the treatment plan and targets - Implement treatment plan, to strengthen emotion regulation and distress tolerance | [14] |
| Cognitive-based therapy for suicide prevention [15, 16] | - Specified session structure; time-limited therapy - Interventions are cognitive or behavioural in nature, and are chosen dependent on cognitive case formulation - Patient and clinician work collaboratively - Clinician delivers all interventions in the context of a warm, non-judgmental, empathetic, collaborative therapeutic setting | - Identify and evaluate automatic thoughts and beliefs - Modify fundamental dysfunctional thoughts and beliefs, through cognitive reappraisal and restructuring, modifying core beliefs, identifying reasons for living, developing coping cards, reducing impulsivity - Implement behavioural strategies (such as increasing pleasurable activities, improving social support, sensory self-soothing) to modify mood and manage overwhelming emotions that do not involve self-harm - Develop and implement a suicide safety plan | [16-18] |

**References**

1. U.S. Department of Health and Human Services (HHS) Office of the Surgeon General and National Action Alliance for Suicide Prevention. 2012 National strategy for suicide prevention: goals and objectives for action*.* Washington, DC: HHS; 2012.
2. Australian Government Department of Health and Ageing. Living Is For Everyone (LIFE) Framework: A framework for prevention of suicide in Australia. Sydney: Commonwealth of Australia; 2008.
3. Japanese Ministry of Health, Labour and Welfare. The general principles of suicide prevention policy: toward the creation of a society where no one is driven to suicide (Cabinet Decision, 28th August 2012). http://jssc.ncnp.go.jp/file/pdf/2015-1101_GeneralPlinciples.pdf. Accessed 20 Jul 2018.
4. National Institute for Health and Clinical Excellence (NICE). Self-harm in over 8s: short-term management and prevention of recurrence. Manchester, United Kingdom: NICE; 2004. https://www.nice.org.uk/guidance/cg16/resources/selfharm-in-over-8s-shortterm-management-and-prevention-of-recurrence-pdf-975268985029. Accessed 05 July 2018.
5. National Institute for Health and Clinical Excellence (NICE). Self-harm in over 8s: long-term management. Manchester, United Kingdom: NICE; 2011. https://www.nice.org.uk/guidance/cg133/resources/selfharm-in-over-8s-longterm-management-pdf-35109508689349. Accessed 05 July 2018.
6. National Institute for Health and Clinical Excellence (NICE). Self-harm. Manchester, United Kingdom: NICE; 2013. Available from https://www.nice.org.uk/guidance/qs34/resources/selfharm-pdf-2098606243525. Accessed 05 July 2018.
7. Jacobs DG, Baldessarini RJ, Conwell Y, Fawcett JA, Horton L, Meltzer H, et al. Practice guideline for the assessment and treatment of patients with suicidal behaviors. Arlington, VA: American Psychiatric Association; 2003.
8. Working Group of the Clinical Practice Guideline for the Prevention and Treatment of Suicidal Behaviour. Clinical practice guideline for the prevention and treatment of suicidal behaviour. Madrid, Spain: Ministry of Health and Social Policy, Galician Health Technology Assessment Agency; 2012.
9. Jobes DA. Managing suicidal risk: a collaborative approach (2nd ed.). New York, NY: Guilford Press; 2016.
10. Andreasson K, Krogh J, Wenneberg C, Jessen HK, Krakauer K, Gluud C, Thomsen RR, Randers L, Nordentoft M. Effectiveness of dialectical behavior therapy versus collaborative assessment and management of suicidality treatment for reduction of self‐harm in adults with borderline personality traits and disorder—a randomized observer‐blinded clinical trial. Depress Anxiety. 2016; doi:10.1002/da.22472.
11. Ellis TE, Rufino KA, Allen JG. A controlled comparison trial of the Collaborative Assessment and Management of Suicidality (CAMS) in an inpatient setting: outcomes at discharge and six-month follow-up. Psychiatry Res. 2017; doi:10.1016/j.psychres.2017.01.032.
12. Ellis TE, Rufino KA, Allen JG, Fowler JC, Jobes DA. Impact of a suicide‐specific intervention within inpatient psychiatric care: the Collaborative Assessment and Management of Suicidality. Suicide Life Threat Behav. 2015; doi:10.1111/sltb.12151.
13. Comtois KA, Jobes DA, O'Connor SS, Atkins DC, Janis K, Chessen CE, et al. Collaborative assessment and management of suicidality (CAMS): feasibility trial for next-day appointment services. Depress Anxiety. 2011; doi:10.1002/da.20895.
14. Asarnow JR, Berk M, Hughes JL, Anderson NL. The SAFETY program: A treatment-development trial of a cognitive-behavioral family treatment for adolescent suicide attempters. J Clin Child Adolesc Psychol. 2015; doi:10.1080/15374416.2014.940624.
15. Wenzel A, Brown GK, Beck AT. Cognitive therapy for suicidal patients: scientific and clinical applications. Washington, DC: American Psychological Association; 2009.
16. Stanley B, Brown G, Brent DA, Wells K, Poling K, Curry J, et al. Cognitive-behavioral therapy for suicide prevention (CBT-SP): treatment model, feasibility, and acceptability. J Am Acad Child Adolesc Psychiatry. 2009; doi:10.1097/CHI.0b013e3181b5dbfe.
17. Brown GK, Ten Have T, Henriques GR, Xie SX, Hollander JE, Beck AT. Cognitive therapy for the prevention of suicide attempts: a randomized controlled trial. JAMA. 2005; doi:10.1001/jama.294.5.563.
18. Tarrier N, Taylor K, Gooding P. Cognitive-behavioral interventions to reduce suicide behavior: a systematic review and meta-analysis. Behav Modif. 2008; doi:10.1177/0145445507304728.
